# Isolation and Determination of Antibacterial Sensitivity Characteristics of *Staphylococcus aureus* from Lactating Cows in West Shewa Zone, Ethiopia

**DOI:** 10.1155/2023/3142231

**Published:** 2023-03-28

**Authors:** Negassa Feyissa, Tesfaye Alemu, Dagim Jirata Birri, Asnake Desalegn, Melaku Sombo, Shubisa Abera

**Affiliations:** ^1^Ambo University, College of Agriculture and Veterinary Science, Department of Veterinary Laboratory Technology, Ethiopia; ^2^Addis Ababa University, College of Natural and Computational Science, Department of Biology, Ethiopia; ^3^National Animal Health Diagnostic and Investigation Center (NAHDIC), Sabata, Ethiopia

## Abstract

*Staphylococcus (S.) aureus* is one of the etiologies of bovine mastitis, hindering milk production and productivity in dairy farms. This study was aimed at assessing the distribution of bovine mastitis and the isolation rate of *S. aureus* in milked cows of West Shewa Zone. The clinical mastitis was diagnosed by physical methods including observation and palpation, whereas the subclinical mastitis was tested by the California mastitis test (CMT). All of the cows tested for mastitis were aseptically sampled (teat-milk) for bacteriology. The bacterium was primarily identified based on colony characterization, catalase, coagulase tests, and Gram stain reaction. Finally, MALDI-TOF Biotyper confirmed the species. The antibacterial sensitivity characteristics of the isolates to different antibacterial drugs were tested by the disk diffusion method. The drugs were selected based on the frequent usage in veterinary medicine in the study area. By using particular primers, the presence of the resistance (*mecA* and *blaZ*), and thermonuclease (*nuc*) genes were determined by polymerase chain reaction (PCR). The data were analyzed by R statistical software. The associations between the dependent variables (prevalence of mastitis and *S. aureus*) and the explanatory variables were analysed by chi-square (*χ*^2^) and logistic regression tests at a 95% confidence interval (CI). Accordingly, 258 lactating cows were examined, of which 97 (37.6%) were mastitis positive. Of these mastitis positive cows, 59 (60.8%) were subclinical and 38 (39.2%) were clinical. Among the 258 milk samples, 43 (16.7%) were positive for *S. aureus*. According to the results of the current investigation, subclinical mastitis was significantly more prevalent than clinical mastitis (*p* < 0.05). The disease was found varied with the lactation stage of the animal, milking with washed hand, udder washing before milking, and tick infestation of the teat. In comparison to animals from farms with lower number of lactating cows, the prevalence of the bacteria was significantly higher in animals managed in farms with large (OR = 12.58, 95% CI = 2.33–68.54, and *p* < 0.05) and medium (OR = 12.58, 95% CI = 2.33–68.54, and *p* < 0.05) population of lactating cows per herd. The isoation rate of the bacterium was also found significantly higher in tick-infested cows (OR = 27.69, 95% CI = 9.71–93.01, and *p* < 0.05) than tick free cows. The antibiogram tests revealed that the isolates resisted penicillin G and tetracycline group drugs (oxytetracycline and tetracycline). Moreover, the nuc gene was confirmed to be present in all of the examined isolates. However, they were not found harboring *blaZ* and *mecA* genes. We concluded that *S. aureus* is sustaining as a main causative agent of bovine mastitis, and they were resistant to the frequently used antibiotics in public and veterinary medicines in the study areas. Therefore, research-based interventions need to be taken in action to combat the pathogen.

## 1. Introduction

The dairy products are among the resources of the livestock sector, which play important domestic economic roles in Ethiopia. However, the production and productivity of milk, which is the raw material for dairy products, are hindered by various factors, among which diseases, feed, and the genetic variety of the animals are the most frequently incriminated [1, 2]. Mastitis has been mentioned among the major obstacles to affect the dairy industry by incuring the dairy farms to unnecessary expenses worldwide in general and in developing countries in particular [3].

According to the symptoms, mastitis can be classified as clinical and subclinical. The clinical and subclinical mastitis are assigned whether the signs of the disease are determined by a tentative diagnosis or not. Although only the abnormalities of the milk (changes in color and/or consistency) might be determined in the early stage of clinical mastitis. However, when the illness worsens, the udder begins to show signs of inflammation, such as swelling, redness, heat, and pain. In subclinical mastitis (SCM), however, only a reduction in milk yield is apparent without obvious observable alteration in milk or udder of the animal; hence, it remains undetected for a long period of time [4]. As a result, SCM has been considered as a more istractive and prevalent type of the disease than clinical mastitis. It has been estimated that SCM alone can cause more than 90% of total milk losses of dairy farms in Ethiopia [2].

Bovine mastitis is caused by either trauma or infections. Bacteria, fungi, and viruses are implicated in causing mastitis in bovines. Among the bacterial pathogens, S. aureus has been detemined to cause mastitis in animals, and food poisoning in dairy products (particularly in yogurt and cheese) [5, 6].

One of the unique characteristics of *S. aureus* in causing mastitis is that it is difficult to cure once it has developed. This might be attributed to the fact that, it possesses a wide variety of virulence and pathogenicity factors including quorum sensing, which enable it to escape immune defense mechanisms, and then causes infections in the availability of immune cells [7]. The other virulence factors include swift development of antibiotic resistance, enterotoxigenicity, and production enzymes such as staphylokinase, lipase, and hemolysins. The antibiotic resistance development nature of the pathogen might be linked to acquiring mobile genetic elements through gene transfer [6, 8]. For example, the methicillin-resistant S. aureus (MRSA) strains of the bacterium have been evolved by acquiring genetic components known as staphylococcal cassette chromosome mec (SCCmec), [9]. SCCmec contains various virulence genes of which mecA, codesfor a modified penicillin-binding protein (PBP-2a). Since PBP-2a has less affinity for methicillin, *mecA positive S. aureus* has developed resistance to methicillin which is drug of choice to treat beta-lactam antibiotic resistant S. aureus infections. In addition the genetic cassette may also contain genes encoding resistance to other groups of antibiotics, and various other virulence factors, including enterotoxins [6, 8]. Nevertheless, penicillin resistance of *S. aureus* might be attributed to penicillinase enzyme production. Penicillinase enzyme, which is coded by blaZ gene,is responsibe for the hydrolysis of penicillin and penicillin derivatives by breaking the -lactam ring of penicillin [10–12]. Such characteristics of the pathogen might lead to fatal infections caused by the strains because of the lack of alternative antibiotics [6, 13].

Furthermore, the thermostability nature of *S. aureus* is linked with the *nuc* gene, which encodes the heat-stable enzyme known as thermonuclease. Therefore, *nuc *gene-positive* S. aureus* can survive in heat-processed foodstuffs, and/or the toxins they produce might be thermostable. However, although the *nuc* gene detection in *S. aureus* has been conducted for species identification [14, 15], the *nuc* gene is not harbored by all strains of *S. aureus* [6, 16].

Therefore, since *S. aureus* is an important disease-causing organism in animals and humans through infection or toxin production in foods [6], assessment of the prevalence and antibiotic susceptibility characteristics of the bacterium in lactating cows can play significant roles in the application of treatment and control procedures against the pathogen. The detection of the genes (*mecA*, *blaZ*, and *nuc*) also helps us to have better understanding of the types of antibiotics intended to be used and/or expect the type of enterotoxins produced (if any) by the isolates. Although few previous studies have been conducted in the study area, they have not detected the resistance or the thermonuclease gene in *S. aureus.* The current study was, therefore, carried out to isolate, identify, and detect resistance genes in *S. aureus*.

## 2. Materials and Methods

### 2.1. Study Area

**Figure 1:**
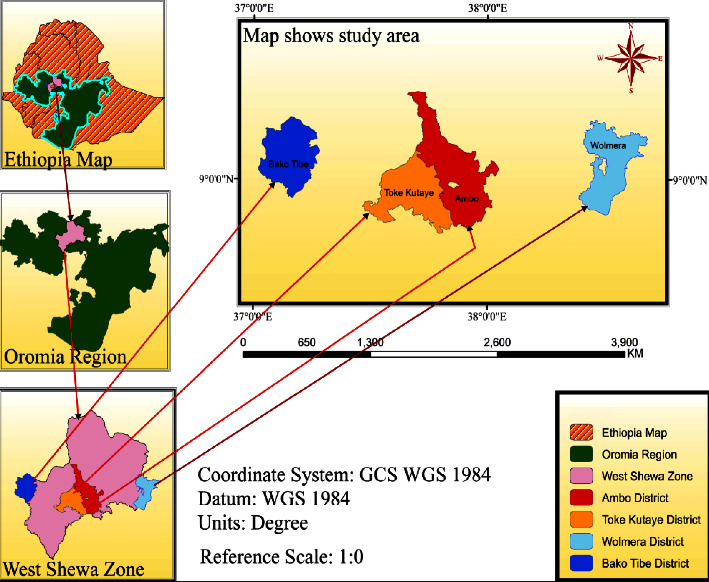
Map of the study area.

West Shewa zone is expected to hold the total population of 2,058,700, of whom 1,028,500 are male and 1,030,200 are female. There are about 1.23 million cattle, 0.43 million sheep, 0.19 million goats, 0.207 million equines, and 1.2 million chickens reared in the zone (Figure 1).

For the current study, four districts of the West Shewa zone, namely, Walmara, Ambo, Toke Kutaye, and Bako Tibe, were randomly selected. The agroecological features, and geographical situations of the districts are illustrated in Table 1.

### 2.2. Study Animal

Inclusion Criteria: the study included lacating cows of the selected districts. That is to be included in the study, the cow should have been lactated at the time samples were collected. Both the local and crossbred cows (crossed between the Zebu and exotic breeds) were included.

Exclusion Criteria: cows, which were not lactating at the sampling time, were excluded from the study.

The ages of the animals were recorded according to the information obtained from the owners. The management systems of the farms were assigned based on the criteria set, and judged by observation.

### 2.3. Study Design

The investigation was conducted between May 2020 and March 2021 using cross-sectional types of the study design.

### 2.4. Sample Size Determination

Based on Thrusfield's [17] earlier formula, the number of animals that were sampled was estimated as follows:

(1) $$\begin{array}{c} N=\frac{z^{2}}{d^{2}}p\left(1-p\right)\text{,} \end{array}$$

where *N* is for sample size, *z* is the *z* value at 95% confidence intervals (1.96), *p* stands for predicted prevalence, and *d* stands for absolute precision, which is 0.05 at 95% CI.

(2) $$\begin{array}{c} N=\frac{1.96^{2}}{0.05^{2}}0.71\left(1-0.71\right)=318\text{.} \end{array}$$

Since the earlier studies performed in some localities of the area reported the prevalence of mastitis to be 70.6%, the number of samples was then calculated with the expected prevalence of 0.71. Therefore, a total of 318 animals were expected to be tested for mastitis. However, only 258 animals were checked for mastitis. The pooled milk samples from all functional teats per animal were collected from all mastitis-checked animals for bacteriology. 

### 2.5. Sampling Techniques

The districts, the peasant associations (PAs) in each district, and the farmers who had lactating cows at the time of sampling were randomly selected using a lottery system. However, since the advanced (semi-intensive and intensive) dairy farms were few in number, all of them were purposefully selected for the study. The clinical- mastitis-positive animals were sampled purposively. However, randomly chosen cows that did not exhibit obvious indications of inflammation on their teats or udders were examined for subclinical mastitis using the California mastitis test (CMT).

The number of milked cows in each farm was divided into three categories as: small (1–5 heads), medium (6–10 heads), and large (>10 heads). The lactating stage of the animals was classified in such a way that “early” from 0 to 3 months, “mid” from 3 to 6 months, and “late” longer than 6 months since gestation. The cows were divided into three age groups as: young < 4 years old, medium from 4 to 6 years, and adults > 7 years old. The cows were also grouped according to their parity level as few (1-2 calving), medium (3–5 calving), and many (>5 calving) [18].

Physical observation and palpation (when needed) of the animal were made to determine the body condition of the animal, tick infestation of the teat area, and presence of clinical mastitis. The body condition of the animal was recorded as good, moderate, or poor according to the criteria set by Metaferia et al. [19]. The clinical mastitis was considered as positive whenever there were changes in color and/or consistency of milk, or the cardinal signs of udder inflammation were observed.

The CMT was carried out in accordance with instructions provided by Quinn et al. [20]. Milk from each functional teats was squirted in each CMT paddle cup and then combined with roughly the equal amounts of 3% CMT reagent. The puddle was moved gently to agitate the mixture. The production of jelly (viscous) consistency within 3–5 minutes was considered CMT positive and then recorded as subclinical mastitis positive.

Strict aseptic practices were applied to prevent contamination from milker hands, the barn surroundings, and skin of the udder and teats. Briefly, the sample collector's hands were protected with gloves and disinfected with 70% ethanol. The teat were washed, disinfected with 70% ethanol, and the foremilk (initial jets) was expelled. A milk sample of 3 to 5 mL was drawn into a sterile universal bottle. The bottle was then labeled (with the animal ID, district, and the date of collection), and transported to the laboratory (Zoonosis and Food Safety Research Laboratory of Ambo University). The milk samples for bacteriology were collected as the pooled sample that milk from all functional quarters of a cow was squirtedinto a single test tube for bacterial identification. Therefore, the prevalence rate of S. aureus was expressed as per cow. 

### 2.6. Isolation and Identification of *S. aureus*

In the laboratory, each sample was enriched with approximately 5 mL of sterile brain heart infusion (BHI) broth and incubated for 24 hours at 37°C. Consequently, the enriched culture was then spread onto a mannitol salt agar (MSA) plate and incubated at 37°C for 24 hours. Bacterial growth and mannitol fermentation within 24 hours were checked. The single colony was chosen and subcultured on brain-heart-infusion (BHI) agar. The pure colony on BHI agar was then stained by Gram stain, and observed under a light microscope. The morphology and arrangements of the bacterial cells was determined. Gram positive (purple blue) cocci with grape-like cellular arrangement were presumptively considered as staphylococci. Primary biochemical tests such as catalase and coagulase tests were carried out on the presumptive isolates [6].

Finally, the suspicious isolates were confirmed as S. aureus by matrix-assisted laser desorption/ionization time-of-flight (MALDI-TOF) Biotyper mass spectrometry using the extended direct transfer (EDT) approach in accordance with the procedure described in Feyissa et al. [6].

### 2.7. Antibiogram Characteristics of *S. aureus* Isolates

To assess the isolates' susceptibility to antibacterial agents, we used disk diffusion techniques [21]. Initially, the purity of the isolates was checked on BHI agar. Second, 3-4 distinct pure colonies (24 hours culture) were suspended in a sterile 0.85% NaCl solution (to a concentration level of 0.5 McFarland turbidity). The turbidity was measured by using the McFarland Densitometer. The cotton swab on wooden stick was deeped into the suspension and streaked onto Muller–Hinton agar plates until the inoculum had sufficiently covered the plate's surface. Using a disk dispenser, the antibiotic disks were then applied to the agar plate and incubated at 37°C for 18 hours. The zones of inhibition of each antibiotic were measured (in mm), and the diameter was compared to the guidelines provided by the CLSI manual [22]. The drugs were chosen based on their availability in the laboratory and distribution in local veterinary clinics and pharmacies. As a result, the following antibiotics were chosen:

1. Erythromycin (15 mg)
2. Trimethoprim/sulfamethoxazole (1.25/23.75 mg)
3. Ciprofloxacin (5 mg)
4. Oxytetracycline (30 mg)
5. Penicillin G (10 units)
6. Cefoxitin (30 mg)
7. Gentamycin (10 mg)
8. Tetracyclin (30 mg)
9. Novobiocin (5 *μ*g)
10. Clindamycin (2 mg).

### 2.8. Antibiotic Resistance and Thermonuclease Gene Determination

#### 2.8.1. Bacterial DNA Extraction

Using the Qiagen DNeasy extraction kit (Thermo Scientific, Germany), the isolates' DNA was extracted in accordance with the manufacturer's instructions [6].

#### 2.8.2. Gene Amplification Using PCR

In the current study, mecA, nuc, and blaZ genes of *S. aureus* isolates were attempted to be amplified by PCR. The procedure used a 31 *μ*l master kit mixture which contains the following ingredients:

1. 2 *μ*L (2 *μ*L *∗* 2 *∗* 3 = 12 *μ*L) each primer
2. *μ*l of 25 mM MgCl_2_
3. 5 *μ*l of PCR buffer
4. 0.5 *μ*l of dNTP
5. 11.8 *μ*l of RNase-free water
6. 0.2 *μ*l of Taq polymerase enzyme

An equal amount of 2 *μ*l of extracted DNA from each sample and 2 *μ*l of the master mix were mixed separately. Sterile water was used as the negative control. The specific primers of *mecA*, *nuc* [15], and blaZ [12] (Eurofins Genomic India Pvt. Ltd) were employed to target the oxacillin-resistant, thermonuclease, and penicillinase genes, respectively (Table 1).

The amplification of the genes was carried out in such a way that the above mixture was added to the PCR tubes and labelled gently. The PCR tubes with all the components were transferred to a Flex cycler thermal cycler (Biometra Gmbh, Germany). The thermal cycler was programmed as initial denaturation 95°C for 5 min and then the final denaturation at 95°C for 30 sec, annealing at 55°C for 30 sec, extension at 72°C for 1 min, and a final extension at 72°C for 10 min repeating for 37 cycles [23].

For the PCR amplification of the genes, the following specific primers were used:

5′GTAGAAATGACTGAACGTCCGATGA (mecA-F) and 5′CCAATTCCACATTGTTTCGGTCTAA3′ (mecA-R) with product length of 163 bp [15].

5′GCGATTGATGGTGATACGGTT (nuc-F) and 5′AGCCAAGCCTTGACGAACTAAAGC (nuc-R) with product length of 279 bp [15].

5′TACAACTGTAATATCGGAGGG (blaZ–F) and 5′CATTACACTCTTGGCGGTTTC (blaZ-R) with product length of 846 bp [11].

#### 2.8.3. Gel Electrophoresis and Visualization of Amplicons

The PCR products that were stained with ethidium bromide were separated by electrophoresis in a 1.5% (w/v) agarose gel. The PCR product mixed with loading dye (bromophenol blue) was applied in the slots of the agarose gel. A maximum of 600 bp of DNA markers (Qiagen; Germany) with 100 bp interval DNA fragments were used to estimate the length of the amplicons. Electrophoresis was then run for one hour at 120 V with 1X TBE buffer. The separated PCR products in electrophoresis were then photographed using UV illumination and recorded by a gel documentation system (Gel DocTM XR+, BioRAD; Germany).

### 2.9. Data Analysis

Data from the field and lab were collected, coded, and entered into Microsoft Excel 2016 files.

R statistical analysis software was used to analyze the data. The association between the dependent variables (the prevalence of mastitis and S. aureus) and the risk factors was determined using the chi-square (*χ*^2^) test and univariable and multivariable logistic regressions at 95% confidence intervals and a *p* value <0.05.

## 3. Results

### 3.1. *S. aureus* Isolation Rate and Risk Factors

A total of 258 milk samples from lactating cows were collected; 97 (37.6%) of these samples were from mastitis-positive cows, of which 59 (60.82%) were subclinical and 38 (39.5%) were clinical cases.

Out of the total 258 milk samples collected, 43 (16.67%) were *S. aureus* positive. The bacterium was isolated at a rate of 34/97 (35.05%) in mastitis-positive animals and 9/161 (5.59%) in mastitis-negative animals. Of the 34 *S. aureus* isolates from mastitis-positive cows, 19 (55.88%) were from clinical and the rest 15 (44.12%) were from subclinical mastitis. The chi-square (*χ*^2^) test analysis revealed a significant correlation between the cows' mastitis positivity and the rate of *S. aureus* isolation. Similarly, the rate was found significantly varied with the type of mastitis that the bacterium was highly significant in clinical mastitis compared with subclinical mastitis (*p* < 0.05). This might make it evident that bovine mastitis which is caused by *S. aureus* is mostly clinical.

The logistic regression analysis of *S. aureus* and associated risk factors is displayed in Table 2. The univariate logistic regression analysis revealed that the districts, farm type, animals' body conditions, number of lactating cows in the herd, washing of the milker's hands and udder before milking, tick infestation, and mastitis positivity were associated significantly with the isolation rates of *S. aureus*. In the analysis of multivariate logistic regression, *S. aureus* was isolated at a significantly higher rate in the animals from farms with a large number of lactating cows in the herd and those infested with ticks than in the animals from farms with a small number of lactating cows and free of tick infestation, respectively (*p* < 0.05).

Also, we made an effort to identify the prevalence of mastitis and other risk variables among lactating cows in the research area. Out of 258 cows examined, 97 (37.6%) had mastitis, with 38 (39.18%) having clinical mastitis and 59 (60.82%) having subclinical mastitis. The occurrence rate of the disease was found to significantly vary with the farm type, lactation stage, milker's hand washing before milking, udder washing before milking, and current tick infestation of the teat. Hence, the occurrence of the disease was significantly higher in mid and late than early lactation stage and in cows managed under an extensive management system than in an intense management system. Furthermore, the disease occurred more frequently in animals milked with unwashed hands than in animals milked with washed hands, in animals whose udders were not washed before milking than in animals washed before milking, and in tick-infested animals than in tick-free animals (*p* < 0.05) (Table 3).

The chi-square test model revealed that SCM was generally significantly higher than clinical mastitis in the population. The disease types were discovered to vary with the age group; current tick infestation of the teat indicated that the animals in the younger age group were developing subclinical mastitis more frequently than the medium-aged groups. Tick-infested teats, on the other hand, were found to develop clinical types of the disease more frequently than tick-free teats (*p* < 0.05).

### 3.2. Antibiogram Characteristics of *S. aureus*

The antibiotic susceptibility profiles of 10 of the 43 *S. aureus* isolates were examined using 9 antibiotic disks. In the current study susceptibility of the isolates to Novobiocin was also determined. However, it has been performed as the default method for identifying *Staphylococcus* species from other Gram-positive cocci. Therefore the drug was used as supplementary test for S. aureus identification methods. The suceptibility test revealed that all the 10 isolates of S.aureus were resistant to Oxytetracycline, Tetracycline, and Penicillin G. In contrast, all of the isolates were shown to be susceptible to the antibiotics Clindamycin, Gentamycin, Ciprofloxacin, Cefoxitin, and Trimethoprim/Sulfamethoxazole (Table 4). Research has also investigated that the antibiotics Penicillin G, ox Tetracyclin, and Trimethoprim/Sulfamethoxazole are the most common drugs used for bovine treatment in the study area.

### 3.3. Antibiotic Resistance and *nuc* Genes

**Figure 2:**
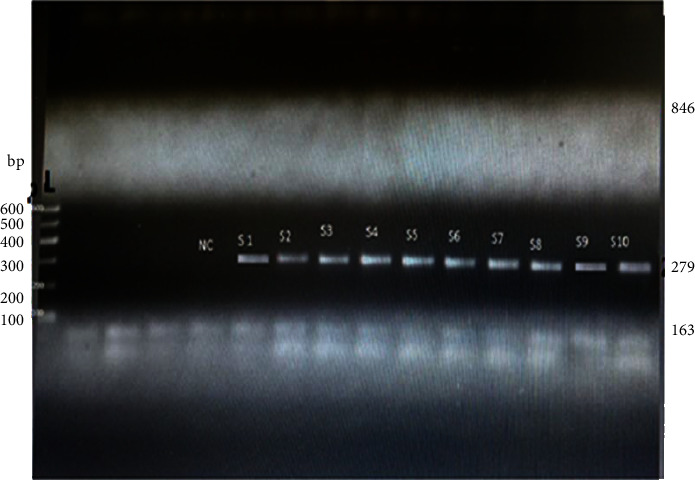
UV-illuminator imaged PCR product after electrophoresis in 1.5% agarose gel. L = ladder, S1–S10 denote samples, and NC stands for negative control. Description: the length of mecA = 163 bp, nuc = 279 bp, and blaZ = 846 bp. Note: bp represents base pairs.

The antibiotic susceptibility tested *S. aureus* isolates were also checked if they have been harboring the drug resistance genes-*blaZ* and *mecA*-e, and thermonuclease (*nuc*) genes. The results of the conventional PCR revealed that all the tested *S.aureus* isolates bore the 279 bp long *nuc* gene. But none of them were found possessing the 163 bp and 846 bp long *mecA* and *blaZ* genes, respectively (Figure 2).

## 4. Discussion

In the current study, *S. aureus* was found in 43 (16.67%) of 258 total milk samples. The rate was significantly higher in mastitis-positive animals (35% versus 5.6%) (*p* < 0.05). Many previous studies have also isolated *S. aureus* at various rates from mastitis-positive cows. This result agreed with Emeru et al. [24] and Girmay et al. [25] reports. However, it is less when compared with the findings of Seyoum et al. [26], Birhanu et al. [2], and Abebe et al. [3], and it exceeded the findings of Bekele et al. [27], Etifu and Tilahun [28], and Lakew et al. [29].

*S. aureus* isolation rates were also found to differ between clinical and subclinical mastitis, with the pathogen being significantly more common in clinical mastitis-positive cows (OR = 2.93) than in subclinical mastitis-positive cows if other variables remained constant. In dissimilarity to the current study, Mekibib et al. [30] and Madut et al. [31] determined a higher *S. aureus* isolation rate in subclinical than clinical mastitis.

*S. aureus* has been identified as one of the primary etiologies of infectious mastitis in cattle [32]. The bacterium resides on superficial surfaces of the udder and the teats, as well as in the teat canal [33–35]. It is among the microflora and hence sometimes infects the animal as an opportunistic pathogen as the consequence of mechanical injury to the teat and other stress factors [36].

In the current study, increase in the number of lactation cows in the herd and tick infestation were linked with the higher isolation rates of *S. aureus*. The number of the lactating cows might affect the hygienic practices such as washing of milker's hands and teats of the animals before milking unless there is an increase in labor resources in the farm. In line with the current findings, Gebremedhin et al. [37] found a direct correlation between tick infestation and the prevalence rate of *S. aureus* in milk samples, as well as an inverse correlation between teat washing before milking and the isolation rate of *S. aureus*. Vouraki et al. [38] also reviewed that there have been reports of *S. aureus* isolation from *Rhipicephalus* spp. tick. *S. aureus* can spread from infected to uninfected udder quarters during milking if the cow's udder or teats are not cleaned before milking since it lives on the udder or teat surface of cows.

The prevalence of *S. aureus* did not significantly vary across animal parity levels in the current investigation (*p* > 0.05). Birhanu et al. [2], Garedew et al. [39], and Tasse et al. [40], however, reported the increment of the isolation rate of *S. aureus* with the parity number of cows.

It has been determined that 37.6% (97/258) of the tested cows were mastitis positive. This result was consistent with that of Sarba and Tola [41], who found that the prevalence of bovine mastitis in the Ambo district was 41.7%. However, Dabele et al. [18] found a higher prevalence rate (30.5%) of the disease in selected districts of the zone. The findings of most of the previous studies regarding bovine mastitis conducted in other parts of the country have reported the prevalence rate ranging between 46% and 73% which were higher than the current finding [3, 23, 25–28, 41, 42].

We found that the animals at the medium and late lactation stages were about 5 and 6 times more mastitic, respectively, than the early lactation stage. Similarly, the management systems were found significantly affecting the prevalence rate of the disease. Accordingly, the cows managed under an extensive farming system were found about 8 times more attacked by mastitis than the cows managed under an intensive farming system. This might be because the extensive management system exposes the animal to trauma and tick infestation during grazing in the field, which, in turn, exposes the teats to pathogenic microorganisms including *S. aureus*. The rate of isolation of *S. aureus* in the current study was higher in tick-infested animals (OR = 27.69, 95% CI: 9.71–93.01, and *p* < 0.001) than in tick free lactating cows. The findings are consistent with previous research on the relationship between tick infestation and mastitis in Ethiopian cattle [43–46]. The positive association between tick infestation and bovine mastitis has been reported from different parts of the world [30, 37, 46, 47]. However, very few research studies, for example, Tolosa et al. [49] and Dabele et al. [18] in Ethiopia, reported that there is no significant variation in prevalence mastitis between tick-infested and tick free cows. Since ticks damage the tissues of teats and can be vectors for pathogenic microorganisms, they are strongly associated with udder lesions and mastitis in cattle [50–52]. However, little has been understood about the association between the parasite infestation and mastitis in Ethiopia [1].

In this research, lactation stage was found associated with bovine mastitis and the finding was in accordance with the previous findings [53, 54]. The finding agrees with Baraki et al. [55], Etifu and Tilahun [28], Abebe et al. [3], and Sarba and Tola [41] reports. According to Abera et al. [42], the rate of bovine mastitis was higher in cows with above 6 months of lactation than the early lactating cows. Similarly, Abebe et al. [3] and Dabele et al. [18] determined that the animals with early lactation stage have less probability to be affected by mastitis than those in the above middle lactation stage. Kitila et al. [45] and Argaw [53] stated, however, that bovine mastitis is more prevalent in cows with early stage than the middle lactation stages.

In our study, we also determined that washing hands before milking played a significant role in reducing mastitis in lactating cows. Our determination agreed with the finding of Shittu et al. [56] who found that the cows in the farms where hand washing before milking had been practiced were less affected by mastitis than the counterpart. Milker's hands might serve as the main vehicle for the spread of pathogenic microorganisms among the teats of the same animal and/or different animals. It is, therefore, crucial that washing hands within the interval of milking each animal can reduce the transmission chances among the animals [3].

Udder washing is an important hygienic practice to reduce not only the contamination of milk by pathogens of animal origin, but it also reduces the distribution of mastitis-causing microorganisms from animal to animal. Washing udders prior to milking can also remove dirt, organic matter, and microorganisms, promote milk let-down, and improve milk quality. The evidence found in this study confirms the practice to reduce the occurrence of mastitis in bovine. Previous studies also pointed that udder washing was found reducing the prevalence rate of mastitis in bovine [18, 30]. In contrast, however, Abebe et al. [3] did not find the udder washing associated with the reduction of mastitis in cows. But it should be underlined that the practice is not solely used to prevent mastitis since mastitis is a multifactorial disease in dairy animals [2].

The costly nature of mastitis in the dairy industry might be attributed to reduction in milk yield and quality and increment of treatment costs [2, 3, 27, 28]. The disease can also affect the responses of the ovarian follicules in cows as a consequence of which the conception rate can significantly decrease [57].

Furthermore, we have found that subclinical mastitis was significantly prevalent than the clinical type of the disease in the study area. About 61% of the mastitis-positive cows were subclinical, and the rest 39% were clinical mastitis (*p* < 0.05). It has been reported that subclinical mastitis is the more common type of the disease than clinical mastitis [2]. In this aspect, the current finding is in accordance with the findings of most studies [29, 57–58]. Argaw [53] estimated the prevalence of clinical and the subclinical mastitis in Ethiopia to be 23 and 85%, respectively. The occurrence rate of clinical mastitis in this study was 14.73% (38/258), which is greater than the rates reported by Abera et al. [42] (10%), Abebe et al. [3] (3.4%), and Bekele et al. [27] (10%). The prevalence rate of subclinical mastitis (26.81%) is less than the reports of Abera et al. [42], Abebe et al. [3], Birhanu et al. [2], Baraki et al. [55], and Bekele et al. [27] who have reported the rates as 36.7%, 59.2%, 40%, 45.5%, and 47%, respectively.

All the 10 isolates checked for the antibiotic susceptibility were determined resistant to the three of the most commonly used drugs in the veterinary drugs in the study area: Tetracycline, Penicillin G, and Oxytetracycline. However, all of the ten *S. aureus* isolates were susceptible to Trimethoprim/Sulfamethoxazole, Gentamycin, Ciprofloxacin, Erythromycin, Clindamycin and. Cefoxitin, The observational analysis revealed that Pen-streptomycine, Penicillin G, Oxytetracycline, and sulfa drugs such as Sulfadimidine and Trimethoprim/Sulfamethoxazole are the most commonly used antibiotics in veterinary medicine. The disadvantages of using the sulfa drug as a treatment in practical applications are that they are needed in high doses and are less effective against inflammation-causing infections. The current study is in line with the assessments of Bekele et al. [27], Seyoum et al. [26], Mekonnen et al. [60], and Girmay et al. [61] who determined majority of mastitis cows isolated *S. aureus* resistant to Oxytetracycline and/or. Penicillin G.

The augmentation of the resistance pattern of the mastitis-causing microbes including *S.aureus* to commonly used antibiotics poses an additional burden on the industry [3]. It should also be underlined that the resistant microorganisms obtained locally can be disseminated to another area through the transportation of food materials.

All the tested isolates have harbored the *nuc* gene. However, the *blaZ* gene was not observed although the isolates were 100% Penicillin G resistant. *BlaZ* gene is responsible for hydrolysing the beta-lactam antibiotics such as penicillin and ampicillin because it encodes the *β*-lactamase enzyme [62]. The lack of *mecA* in the isolates was confirmation for susceptibility of the *S. aureus* isolates to cefoxitin, and the availability of *nuc* indicated that the isolates were thermostable [62].

## 5. Conclusions

Based on the study's findings, we deduced that *S. aureus* continues to be the primary cause of mastitis in dairy cows. Clinical mastitis was shown to have a greater prevalence of *S. aureus* than subclinical mastitis. Also, it was discovered that the study area has a high distribution of bovine mastitis. The antibacterial susceptibility traits of *S. aureus* showed us that they were extremely resistant to the locally prevalent antibiotics used in veterinary cares. There may be other genetic components or methods by which the bacteria resist penicillin, which could account for the absence of the blaZ gene in the examined *S. aureus* isolates. Therefore, the prevention and control of the pathogen, and as a consequence, the reduction of the prevalence of the disease, need to be designed. Antibiotics with better treatment responses should also be introduced to the area. Furthermore, the penicillin-resistant mechanism of *S. aureus* should be studied in depth.

## Figures and Tables

**Table 1:** General characteristics and agroecological features of the selected districts.

Characteristics	Districts			
	Wolmera	Ambo	Toke Kutaye	Bako Tibe
Administrative town	Holeta	Ambo	Guder	Bako
Distance from Addis Ababa in km	34	114	123	260
Direction	West	West	West	West
Latitude	8°50′–9°15′N	8°47′–9°21′N	9°11′N–9°18′N	9°06′N
Longitude	38°25′–38°45′E	37°32′–38°3′E	38°20′E−38°33′E	37°09′E
Average temperature (range) in °C	16 (9.13–19.66)	22 (15–29)	22 (16–29)	29 (28–31)
Altitude range in masl	2060–3380	1800–3200	1600–3194	1570–2600
Rain fall in mm	1,100	900	900	887

**Table 2:** Study of *S. aureus* isolation rate and related risk factors using binomial logistic regression.

Characteristics	Category	Samples	*S. aureus* positive (%)	Univariate		Multivariate	
				OR (95% CI)	*p* value	OR (95% CI)	*p* value
Districts	Toke kutaye	51	4 (7.84)	1.0		1.0	
	Wolmera	77	18 (22.22)	3.36 (1.16–12.20)	0.039	3.85 (0.92–19.96)	0.081
	Ambo	81	16 (20.78)	3.08 (1.05–11.30)	0.057	3.20 (0.66–18.40)	0.164
	Bako tibe	49	5 (10.20)	1.34 (0.33–5.70)	0.681	0.92 (0.14–6.10)	0.931

Agroecology	Lowland	49	5 (10.20)	1.0			
	Midland	128	20 (15.62)	1.63 (0.61–5.14)	0.358		
	Highland	81	18 (22.22)	2.51 (0.92–8.07)	0.089		

Breeds	Cross	115	21 (14.69)	1.0			
	Local	143	22 (19.13)	1.37 (0.71–2.66)	0.342		

Farm types	Intensive	27	1 (3.70)	1.0		1.0	
	Semiintensive	49	36 (12.24)	3.63 (0.58–70.5)	0.245	0.89 (0.08–23.35)	0.930
	Extensive	182	36 (19.78)	6.41 (1.3–116.3)	0.073	1.77 (0.22–40.61)	0.642

Age group of the animal	Adult	87	17 (13.93)	1.0			
	Medium	122	16 (18.39)	1.39 (0.66–2.95)	0.385		
	Young	49	10 (20.41)	1.58 (0.65–3.71)	0.297		

Lactation stage	Late	67	8 (11.94)	1.0			
	Mid	95	17 (17.89)	1.61 (0.67–4.17)	0.304		
	Early	96	18 (18.75)	1.70 (0.71–4.39)	0.246		

Parity level	Medium	167	24 (14.37)	1.0			
	Few	86	18 (20.93)	1.58 (0.79–3.09)	0.186		
	Many	5	1 (20.00)	1.49 (0.07–10.61)	0.727		

Animal's body condition	Moderate	47	22 (12.43)	1.0		1.0	
	Poor	34	11 (23.40)	2.15 (0.93–4.77)	0.063	1.20 (0.33–4.24)	0.778
	Good	177	10 (29.41)	2.94 (1.20–6.86)	0.014	2.67 (0.69–10.41)	0.152

Number of lactating cows in the herd	Few	224	33 (14.73)	1.0			
	Medium	16	4 (25.00)	1.93 (0.52–5.93)	0.279	9.99 (1.70–57.56)	0.009
	Large	18	6 (33.33)	2.89 (0.95–8.02)	0.047	12.58 (2.33–68.54)	0.003

Washing hands before milking	Yes	154	16 (10.39)	1.0		1.0	
	No	104	27 (25.96)	3.02 (1.55–6.07)	0.001	0.46 (0.11–1.69)	0.262

Washing udder before milking	Yes	158	12 (7.59)	1.0		1.0	
	No	100	31 (31.00)	5.47 (2.71–11.68)	0.000	2.12 (0.50–9.04)	0.304

Current tick infestation of the teat	No	208	11 (5.29)	1.0		1.0	
	Yes	50	32 (64.00)	31.84 (14.2–76.7)	0.000	27.69 (9.71–93.01)	0.000

Mastitis	Negative	161	9 (5.59)	1.0		1.0	
	Positive	97	34 (35.05)	9.11 (4.29–21.24)	0.000	2.34 (0.53–10.86)	0.263

OR = odds ratio and 1.0 = reference group.

**Table 3:** Binomial logistic regression analysis of association between bovine mastitis and risk factors.

Characteristics	Category	Mastitis positive (%)	Univariate		Multivariate	
			OR (95% CI)	*p* value	OR (95% CI)	*p* value
Districts	Wolmera	28 (34.57)	1.0			
	Ambo	33 (42.86)	1.42 (0.75–2.71)	0.285		
	Toke kutaye	19 (37.25)	1.12 (0.54–2.33)	0.754		
	Bako tibe	17 (34.69)	1.01 (0.47–2.11)	0.988		

Agroecology	Highland	28 (34.57)	1.0			
	Midland	52 (40.62)	1.30 (0.73–2.32)	0.381		
	Lowland	17 (34.69)	1.01 (0.47–2.11)	0.988		

Breeds	Local	42 (36.52)	1.0			
	Cross	55 (38.46)	1.09 (0.65–1.81)	0.749		

Farm types	Intensive	4 (14.81)	1.0		1.0	
	Semiintensive	18 (36.73)	3.34 (1.07–12.75)	0.051	2.45 (0.31–22.64)	0.40672
	Extensive	75 (41.21)	4.03 (1.48–14.17)	0.013	8.20 (1.33–68.25)	0.03451

Age group of the animal	Adult	43 (35.25)	1.0			
	Medium	33 (37.93)	1.12 (0.63–1.99)	0.691		
	Young	21 (42.86)	1.38 (0.70–2.71)	0.353		

Lactation stage	Early	28 (29.17)	1.0		1.0	
	Mid	41 (43.16)	1.84 (1.02–3.38)	0.045	5.03 (1.48–19.27)	0.01275
	Late	28 (41.79)	1.74 (0.91–3.37)	0.096	6.12 (1.55–27.32)	0.01229

Parity level	Moderate	51 (30.54)	1.0		1.0	
	Few	43 (50.00)	2.27 (1.33–3.90)	0.003	2.32 (0.78–7.16)	0.13117
	Many	3 (60.00)	3.41 (0.55–26.49)	0.186	0.12 (0.00–2.80)	0.19550

Animal's body condition	Moderate	57 (32.20)	1.0		1.0	
	Good	17 (50.00)	2.11 (1.00–4.45)	0.049	0.64 (0.12–3.20)	0.58828
	Poor	23 (48.94)	2.02 (1.05–3.89)	0.035	1.14 (0.30–4.22)	0.84861

Number of lactating cows in the herd	Small	80 (35.71)	1.0			
	Medium	7 (43.75)	1.40 (0.48–3.90)	0.520		
	Large	10 (55.56)	2.25 (0.85–6.11)	0.101		

Hand washing before milking	Yes	18 (11.69)	1.0		1.0	
	No	79 (75.96)	23.88 (12.6–47.8)	0.000	15.98 (5.6–51.5)	0.0000

Udder washing before milking	Yes	14 (8.86)	1.0		1.0	
	No	83 (83.00)	50.22 (24.4–111.3)	0.000	36.90 (12.2–135.6)	0.0000

Current tick infestation of the teat	No	55 (26.44)	1.0		1.0	
	Yes	42 (84.00)	14.60 (6.77–35.34)	0.000	34.24 (5.9–253.3)	0.00020

*S. aureus*	Negative	63 (29.30)	1.0		1.0	
	Positive	34 (79.1)	9.11 (4.29–21.24)	0.000	1.15 (0.22–6.09)	0.86272

OR = odds ratio and 1.0 = reference group.

**Table 4:** Antibiogram characteristics of *S. aureus*.

Antibacterials	Total tested	Susceptibility		
		Susceptible no (%)	Intermediate no (%)	Resistant no (%)
Ciprofloxacin	10	10 (100)	00 (0.00)	00 (0.00)
Penicillin G	10	00 (0.00)	00 (0.00)	10 (100)
Trimethoprim/sulfamethoxazole	10	10 (100)	00 (0.00)	00 (0.00)
Cefoxitin	10	10 (100)	00 (0.00)	00 (0.00)
Oxytetracycline	10	00 (0.00)	00 (0.00)	10 (100)
Gentamycin	10	10 (100)	00 (0.00)	00 (0.00)
Erythromycin	10	10 (100)	00 (0.00)	00 (0.00)
Tetracycline	10	00 (0.00)	00 (0.00)	10 (100)
Clindamycin	10	10 (100)	00 (0.00)	00 (0.00)
Novobiocin	10	10 (100)	00 (0.00)	00 (0.00)

## Data Availability

The data used to support the findings of the study are available on request from the corresponding author.
